# The protective role of carnosine against type 2 diabetes‐induced cognitive impairment

**DOI:** 10.1002/fsn3.4077

**Published:** 2024-03-12

**Authors:** Qian Wang, Nicholas Tripodi, Zachary Valiukas, Simon M. Bell, Arshad Majid, Barbora de Courten, Vasso Apostolopoulos, Jack Feehan

**Affiliations:** ^1^ Institute for Health and Sport, Victoria University Melbourne Australia; ^2^ Sheffield Institute for Translational Neuroscience, Sheffield University Sheffield UK; ^3^ STEM college, RMIT University Melbourne Victoria Australia; ^4^ School of Clinical Sciences Monash University Melbourne Victoria Australia; ^5^ Australian Institute for Musculoskeletal Sciences, Immunology Program, Western Health The University of Melbourne and Victoria University Melbourne Victoria Australia

**Keywords:** carnosine, cognitive impairment, dementia, type 2 diabetes mellitus

## Abstract

The morbidity and mortality associated with type 2 diabetes mellitus (T2DM) have grown exponentially over the last 30 years. Together with its associated complications, the mortality rates have increased. One important complication in those living with T2DM is the acceleration of age‐related cognitive decline. T2DM‐induced cognitive impairment seriously affects memory, executive function, and quality of life. However, there is a lack of effective treatment for both diabetes and cognitive decline. Thus, finding novel treatments which are cheap, effective in both diabetes and cognitive impairment, are easily accessible, are needed to reduce impact on patients with diabetes and health‐care systems. Carnosine, a histidine containing dipeptide, plays a protective role in cognitive diseases due to its antioxidant, anti‐inflammation, and anti‐glycation properties, all of which may slow the development of neurodegenerative diseases and ischemic injury. Furthermore, carnosine is also involved in regulating glucose and insulin in diabetes. Herein, we discuss the neuroprotective role of carnosine and its mechanisms in T2DM‐induced cognitive impairment, which may provide a theoretical basis and evidence base to evaluate whether carnosine has therapeutic effects in alleviating cognitive dysfunction in T2DM patients.

## Graphical abstract

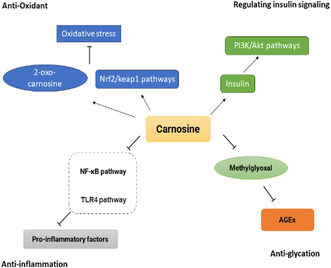

## 1 INTRODUCTION

Type 2 diabetes mellitus (T2DM), a prevalent metabolic disease, has become the seventh leading cause of death worldwide with the exponential rise of obesity (Glovaci et al., 2019; Guariguata et al., 2014; Nanda et al., 2022). In the last 30 years, the morbidity and mortality of T2DM have doubled (Nanda et al., 2022), with the expected number of people with diabetes to reach 592 million by 2035, alongside increased key risk factors such as excess weight and obesity (Ghanbari‐Gohari et al., 2022; Guariguata et al., 2014). Additionally, adding to the burden of diabetes are its complications, such as cardiovascular, diabetic renal disease, retinopathy, neuropathy, as well as cognitive impairment or dementia (Cole & Florez, 2020; Harris et al., 2020). Importantly among these, T2DM induces cognitive impairment and dementia, affecting memory, executive function, further increasing the financial cost of care, and worsening patient outcomes, leading to poor quality of life, and even greater mortality (Cole & Florez, 2020; Zheng et al., 2018; Zilliox et al., 2016). T2DM is strongly associated with risk of dementia, and deficits in attention, processing and motor speed, executive function, and verbal memory (Zilliox et al., 2016). Diabetes is one of 12 key modifiable risk factors that have been identified to contribute to dementia and their treatment could prevent or delay the onset of up to 40% of dementias (Livingston et al., 2020). Therefore, effective control of T2DM has the potential to have a huge impact on dementia.

While the pathological mechanisms underpinning this are incompletely understood, T2DM causes a range of physiological changes which influence the central nervous system (CNS). T2DM causes significant microvascular and macrovascular complications, including neuropathy and cerebrovascular disease (Cade, 2008). Macrovascular disease can lead to stroke and microvascular disease‐induced ischemia and functional hyperemia, leading to cognitive impairment and dementia (Cade, 2008). Importantly, it is not T2DM alone, but rather metabolic, morphological, and functional changes induced by hyperglycemia and insulin resistance which cause cognitive impairment and dementia (Arnold et al., 2018; Barber et al., 2021; Jayaraman & Pike, 2014; Tan et al., 2021). In fact, T2DM‐induced insulin resistance, systemic inflammation, neuroinflammation, oxidative stress, and advanced glycation end product (AGE) accumulation, are the main pathogenic factors thought to result in cognitive decline (Verdile et al., 2015; Zilliox et al., 2016). Insulin resistance is thought to contribute to the progression of dementia through different mechanisms, including promotion of disease‐specific pathological lesions, such as medial temporal lobe atrophy, increased neuronal vulnerability, and neurodegeneration, with subsequent development of amyloid β (Aβ) plaques, tau phosphorylation, neurofibrillary lesions, and α‐synuclein lesions (Mullins et al., 2017). Aβ reduces the influence of insulin on mitochondrial function in the synaptic terminal, diminishing the energy reserves for synaptic plasticity, learning, and memory (Heras‐Sandoval et al., 2012). Additionally, insulin resistance results in a series of immune responses that exacerbate the inflammatory state (Mullins et al., 2017). In T2DM, fatty acids enter the CNS and activate the immune system through the toll‐like receptor 4 (TLR4) protein, causing astrocytes to secrete pro‐inflammatory cytokines (Obadia et al., 2022). Additionally, AGEs interact with their receptors (RAGEs) and generate reactive oxygen species (ROS), causing increased oxidative stress and accumulation of free radicals (Y. Li et al., 2018). Free radicals damage DNA, proteins, and lipids, leading to advancing brain tissue damage (Y. Li et al., 2018). Therefore, insulin resistance, oxidative stress, neuroinflammation, and AGE accumulation are considered as important contributors to T2DM‐induced cognitive impairment (Table 1).

**TABLE 1:** Potential diabetic pathologic changes promote dementia.

Pathologic changes	Mechanisms
Microvascular complications	Impaired blood flow leading to ischemia and functional hyperemia, resulting in cognitive impairment and dementia
Macrovascular complications	Increased risk of stroke and further cognitive decline
Hyperglycemia and insulin resistance	Promotion of disease‐specific pathologic lesions, such as medial temporal lobe atrophy, neuronal vulnerability, and neurodegeneration
Systemic and neuroinflammation	Activation of immune responses including astrocytes, through TLR4 activation, leading to pro‐inflammatory cytokine secretion in the brain to cause cognitive impairment
Oxidative stress	Accumulation of free radicals, causing DNA, protein, and lipid damage
AGEs	AGEs interact with their receptors, generating ROS, and leading to oxidative stress and brain tissue damage
Aβ plaques	Insulin resistance reduces the impact of insulin on mitochondrial function, contributing to the accumulation of Aβ plaques and impaired synaptic plasticity
Tau phosphorylation	Insulin resistance is associated with tau protein hyperphosphorylation, leading to the formation of neurofibrillary tangles and neuronal dysfunction
Brain tissue damage	Insulin resistance and related processes contribute to neuronal damage and loss, a significant factor in dementia development

Abbreviations: Aβ, amyloid β; AGEs, advanced glycation end‐products; TLR4, toll‐like receptor 4; ROS, reactive oxygen species.

Currently, there are no established clinical treatments specifically targeted at cognitive impairment induced by T2DM, but some interventions show promise in alleviating cognitive impairment in these patients. These include exercise, intensive glycemic control, and dietary or nutritional interventions. Therapeutic exercise may attenuate mild cognitive impairment (MCI), with a beneficial effect on the cognitive health of T2DM patients and improving brain structure and function (Callisaya & Nosaka, 2017). However, a study found that a 24‐month moderate‐intensity exercise program had no beneficial effect on cognitive function, largely due to the challenge of sustaining moderate‐intensity exercise among older adults (Sink et al., 2015). Intensive glycemic control is the mainstay of management and prevention of all diabetic complications, aiming to maintain glycated hemoglobin (HbA1c) at 6.5%–7.0% (48–53 mmol/mol) or below (Rodriguez‐Gutierrez et al., 2019). While effective glycemic control has been shown to reduce the rate of brain atrophy, it does not improve cognitive function (Launer et al., 2011). Nutritional interventions, such as the ketogenic diet and vitamin D3 supplementation, have shown promise in protecting neurons and preventing cognitive impairment in T2DM (Bai et al., 2023; Tan et al., 2021). However, these interventions have limitations, including adherence difficulties and unclear mechanisms of action. More recently, intranasal insulin therapy has been applied to patients with type 1 diabetes mellitus (T1DM) and Alzheimer's disease (AD), which may possibly facilitate a reduction in tau phosphorylation and amyloid plaque density that could attenuate cognitive decline (Rdzak & Abdelghany, 2014). However, its efficacy in modulating the level of glucose was limited, proposed to be due to insulin being unable to effectively enter the cerebral circulation, rendering it ineffective (Gancheva et al., 2015). Modulating the effect that diabetes has on cognitive performance may need a multidomain approach in which factors such as blood pressure and weight management are also targeted as this type of intervention has been shown to have the greatest effect in elderly populations (Ngandu et al., 2015). Thus, finding novel treatments, which are cheap, effective, and widely available are required to help reduce the impacts on patients with T2DM and healthcare systems more broadly.

Carnosine, a naturally occurring dipeptide, is composed of β‐alanine and l‐histidine, with biological functions including antioxidant activity, anti‐inflammation, anti‐glycation, antitumor, and antiaging effects (Boldyrev et al., 2013). Carnosine is abundant in skeletal and cardiac muscle as well as brain tissue, where it is synthesized by hydrolysis of endogenous carnosine synthase (CARNS1) (Boldyrev et al., 2013). The two precursors of carnosine, β‐alanine and l‐histidine are easily transported across the blood–brain barrier through amino acid transporters, allowing for carnosine synthesis in the brain (Hawkins et al., 2006). While carnosine can also cross the blood–brain barrier, most of it is synthesized locally in the brain (Jin et al., 2005). Previous work has shown that after administration, carnosine reaches peak concentration in the brain after 6 h, with a different pharmacokinetic curve compared to the blood, suggesting that cerebral carnosine is mainly resynthesized in glial cells (Guliaeva et al., 1989). Glial cells, especially oligodendrocytes, are the primary site of carnosine synthesis in the brain, while neurons and astrocytes are the primary users of carnosine (Berezhnoy et al., 2019). To achieve neuroprotective effects, high concentrations of carnosine must be maintained in brain tissue (Lopachev et al., 2022). This can be accomplished by enhancing the efficiency of glial cells to produce endogenous carnosine and facilitate its transportation into neurons, or through exogenous supplementation of carnosine to increase its concentration within neurons (Lopachev et al., 2022). Carnosine has been shown to have a beneficial effect on cognitive impairment by suppressing neuronal cell death and inflammatory responses in cognition‐related diseases including stroke, AD, and vascular dementia (Artioli et al., 2019). Due to its effect on glucose metabolism, carnosine is also involved in the regulation of blood glucose and insulin resistance in patients with diabetes (Houjeghani et al., 2018). However, the effects of carnosine on T2DM‐induced cognitive impairment and related mechanisms are not fully understood.

This article aims to review the role of carnosine in T2DM‐induced cognitive impairment, and its potential mechanisms, including antioxidant activity, anti‐inflammatory effects, anti‐glycation, and regulating insulin resistance in central nervous system (CNS).

## 2 WHAT IS CARNOSINE?

Carnosine was first discovered in the early 1900s by Gulewitsch and Amiradzbi in Russia (Gulewitch & Amiradzibi, 1900). Carnosine is composed of β‐alanine and l‐histidine, detected in skeletal and cardiac muscle as well as brain tissue (Hipkiss, 2009). Carnosine is one of a number of histidine containing dipeptides (HCDs) which share some physiological characteristics, including anserine, ophidine (balenine), homocarnosine, and acetyl‐carnosine, which are detected in different tissues of mammals including the olfactory bulb, skeletal muscle, the choroid plexus, cerebral cortex, kidneys, the spleen, cerebrospinal fluid, and plasma (Boldyrev et al., 2013). Carnosine is particularly abundant in human skeletal muscle, cardiac muscle, kidneys, and the brain to maintain healthy bodily function (Artioli et al., 2019; Wu et al., 2013). The prevalence of HCDs within distinct tissues underscores their essential roles and specialized functions, which protect myocardial function, enhance cognition, prevent chronic diseases, and collectively overall bodily well‐being (Boldyrev et al., 2013).

### 2.1 Chemical and biochemical properties of carnosine

#### 2.1.1 Antioxidant activity

**FIGURE 1:**
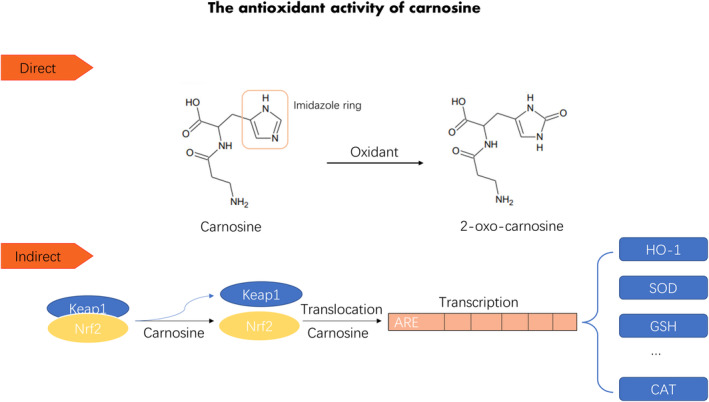
The antioxidant activity of carnosine. CAT, catalase; GSH, glutathione; HO‐1, Heme oxygenase‐1; Keap1, Kelch‐like ECH‐associated protein 1; Nrf2, Nuclear factor‐erythroid factor 2‐related factor 2; SOD, superoxide dismutase.

The antioxidant effect of carnosine is well‐known and has been demonstrated across many chronic diseases (Ahshin‐Majd et al., 2016; Alsheblak et al., 2016; Deng et al., 2018). Carnosine imparts its antioxidant effect by both directly scavenging free radical and oxidizing species, as well as indirectly activating the endogenous antioxidant system via the nuclear factor‐erythroid factor 2‐related factor 2 (Nrf2) pathway. The imidazole ring of carnosine is responsible for its direct ROS scavenger—playing a protective effect against hypochlorous acid—one of the most important biological ROS (Boldyrev et al., 2013). When carnosine reacts with hypochlorous acid, the imidazole ring transfers to imidazole chloramines, thereby reducing oxidative damage (Pattison & Davies, 2006). Additionally, under H_2_O_2_ exposure, carnosine is oxidized to 2‐oxo‐carnosine by its imidazole ring in SH‐SY5Y human neuroblastoma cells while expressing CARNS1 (Ihara et al., 2019). Moreover, 2‐oxo‐carnosine displays stronger antioxidant properties than the corresponding carnosine and glutathione (GSH, an endogenous antioxidant) (Ihara et al., 2019; Kasamatsu et al., 2021). The removal of 2‐oxo‐carnosine from carnosine standards resulted in a significant reduction in antioxidant capacity, which suggests that 2‐oxo‐carnosine is a major driver of the antioxidant activity of carnosine (Figure 1) (Ihara et al., 2019; Kasamatsu et al., 2021).

Not only does carnosine possess direct antioxidant ability, it also indirectly counteracts oxidative damage via the Nrf2 signaling pathway. Following activation, Nrf2 translocates to the nucleus, binding DNA, and triggers antioxidant responses by facilitating various gene products (Li & Kong, 2009). Thus, Nrf2 is considered to be the major transcription factor involved in the induction of antioxidant genes (Li & Kong, 2009; Xiong et al., 2021). Carnosine potentiates the antioxidant capacity of intestinal stem cells by mediating Kelch‐like ECH‐associated protein 1 (Keap1)/Nrf2 signaling, which promotes the intestinal epithelial regeneration response to deoxynivalenol insult (Zhou et al., 2021). In the CNS, carnosine restores the decline of nuclear Nrf2 expression, by ameliorating the increase of malondialdehyde (a marker of lipid peroxidation) and promotes the decrease of GSH and superoxide dismutase (SOD) by Nrf2/Heme oxygenase‐1 (HO‐1) cascade to promote the antioxidant response, which attenuates cognitive impairment in T1DM rats (Ahshin‐Majd et al., 2016; Alsheblak et al., 2016). Carnosine also mediates the HO‐1/HSP 72 (inducible from HSP 70) signaling pathway to alleviate the neuronal damage induced by oxidative stress in animal models of aging (Davinelli et al., 2013). Meanwhile, ROS production is reduced by the suppression of phosphoinositide‐3 kinase (PI3K)/protein kinase B (Akt) pathways activating Nrf2 in mouse podocyte cells following hyperglycemic injury, indicating that carnosine might activate the Nrf2 pathway by modulating insulin signaling pathways to induce a stimulated antioxidant response (Figure 1) (Zhao et al., 2019).

#### 2.1.2 Anti‐inflammatory effects

**FIGURE 2:**
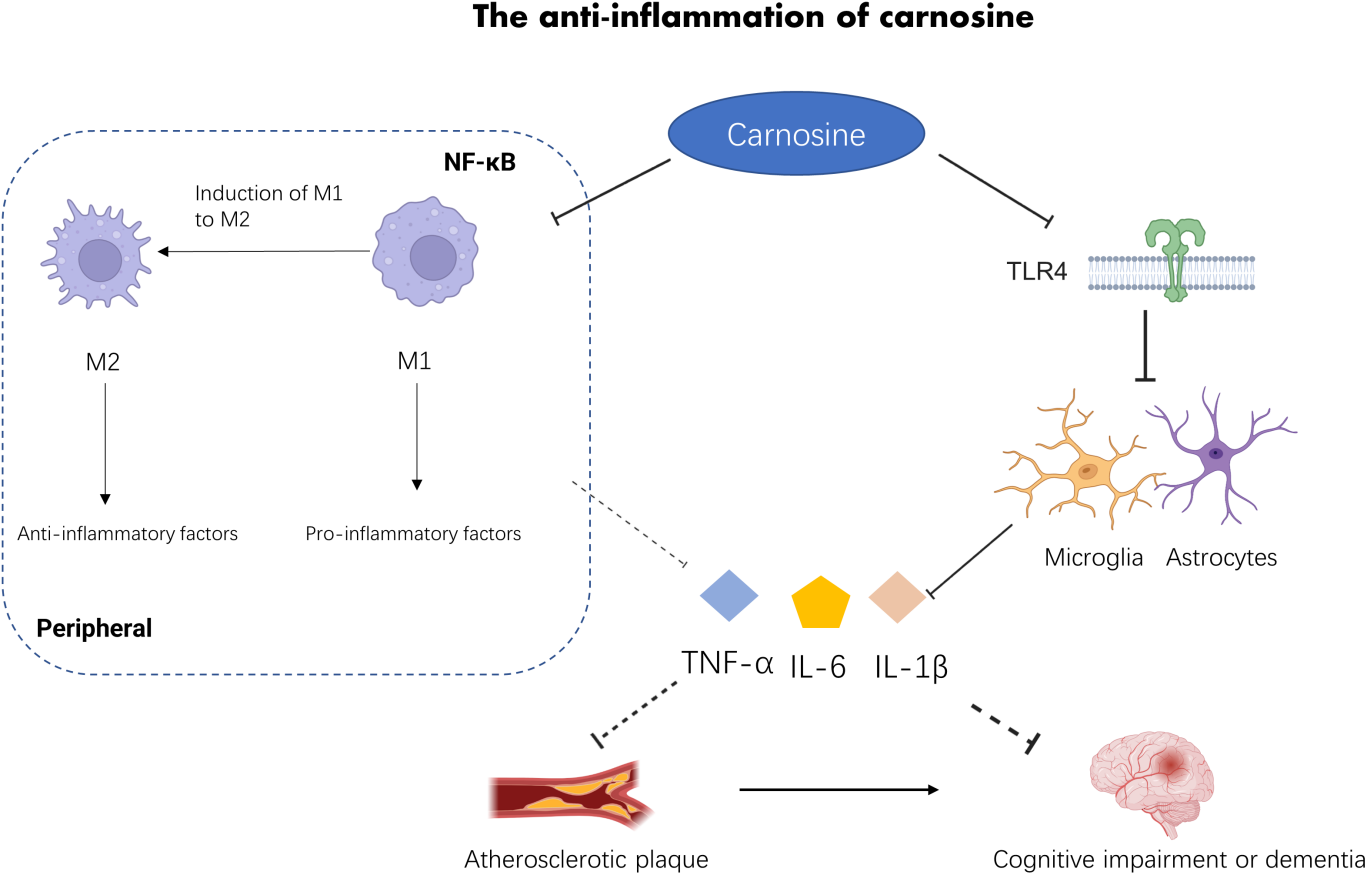
The anti‐inflammation of carnosine. IL‐6, interleukin‐6; IL‐1β, interleukin‐1β; M1, M1 macrophages; M2, M2 macrophages; TLR4, toll‐like receptor 4; TNF‐α, tumor necrosis factor alpha.

In addition to its antioxidative effects, carnosine also has the capacity to directly modulate the immune system with anti‐inflammatory effects. Carnosine demonstrates strong immunomodulatory regulation on macrophages (Caruso, Fresta, Fidilio, et al., 2019), with an in vitro study illustrating that pretreatment with carnosine can attenuate Akt phosphorylation, decrease tumor necrosis factor alpha (TNF‐α) and interleukin (IL)‐6 mRNA levels, and increase IL‐4, IL‐10, and transforming growth factor‐β mRNA levels in phorbol 12‐myristate 13‐acetate (PMA)‐induced RAW 264.7 cells. This suggests that carnosine might promote M1 to M2 macrophage transition, reducing pro‐inflammatory cytokines and increasing production of anti‐inflammatory cytokines (Figure 2) (Caruso, Fresta, Fidilio, et al., 2019). In addition to hyperglycemia, T2DM is characterized by chronic inflammation, which drives many of its associated complications, including cognitive impairment. Carnosine supplementation reduces the levels of pro‐inflammatory cytokines in T2DM, with likely follow effects in the CNS (Yang et al., 2018). Carnosine also exhibits an anti‐inflammatory effect in the context of diabetic complications reducing nuclear factor κB (NF‐κB) signaling and levels of pro‐inflammatory factors in T1DM‐induced nephropathy and T2DM‐induced osteoarthritis (Liu et al., 2020; Yang et al., 2018). Additionally, podocyte inflammation and pyroptosis are suppressed through caspase‐1 silencing following carnosine treatment in diabetic nephropathy (Zhu et al., 2021). This evidence suggests that carnosine acts as an anti‐inflammatory agent in T2DM and its complications.

In the CNS, carnosine may also play an anti‐inflammatory role, mitigating neuronal damage in cognitive disorders. For example, carnosine reduces levels of pro‐inflammatory factors including IL‐6, TNF‐α, cyclooxygenase 2, and TLR4 as well as neuronal cell death in hypothalamic neuronal cells, suggesting anti‐inflammatory action (Kubota et al., 2020). The proposed mechanism underlying these results is that carnosine inhibits the activation of the stress‐activated protein kinase/c‐Jun‐N‐terminal kinase (JNK) signaling pathway which is associated with inflammatory response (Kubota et al., 2020). Carnosine also counteracts the release of pro‐inflammatory cytokines such as IL‐1β induced by Aβ oligomers in microglia and rescues anti‐inflammatory cytokine IL‐10 levels to suppress neuroinflammation and cognitive deficits (Caruso, Fresta, Musso, et al., 2019). Analogous results in animal studies show that activated microglia and astrocytes are inhibited by carnosine treatment in chronic cerebral hypoperfusion and subcortical ischemic vascular models of dementia (Ma et al., 2012; Ma et al., 2018; Xie et al., 2017). Astrocyte reactivity plays a pivotal role in connecting Aβ with initial tau pathology, with activated astrocytes make patients more susceptible to AD pathology (Bellaver et al., 2023). IL‐1β is an important activator of astrocytes, and the inhibitory action of carnosine on IL‐1β upregulation could potentially slow the progression of dementia and cognitive impairment (Caruso, Fresta, Musso, et al., 2019; Ma et al., 2019; Sama et al., 2008). Based on this evidence, carnosine anti‐inflammatory physiologies may improve peripheral chronic inflammation, reduce the release of pro‐inflammatory factors, and rescue the levels of anti‐inflammatory factors in the CNS, thereby ameliorating neuronal damage and cognitive impairment in T2DM (Figure 2).

In cardiovascular diseases (CVD), carnosine prevented early atherosclerotic lesion formation in a high‐fat diet with ApoE^−/−^ mice, with the suggested mechanism being that carnosine suppresses oxidized low‐density lipoprotein‐induced macrophage apoptosis (Barski et al., 2013). Consistent with this, similar animal research shows that carnosine reduces lesion size and promotes plaque phenotype stability, accompanied by decrease of macrophages which facilitate plaque stability in early stage. This decline is correlated with the clearance of apoptotic cells, which efficiently reduces lesion cellularity and the production of pro‐inflammatory factors (Menini et al., 2012). Previous systematic reviews have also shown that carnosine inhibits IL‐6 release from activated macrophages which decreases CVD risk and might be a potential therapeutic to prevent atherosclerotic plaque formation by its anti‐inflammatory properties (Figure 2) (Caruso et al., 2020).

#### 2.1.3 Metal chelating activity

Carnosine chelates a number of metal cations (Cu^2+^, Zn^2+^, Ni^2+^, and Co^2+^) to form a coordination complex, which play essential roles in various biological activities and pharmacological applications (Baran, 2000). Metal cations are also critical for the stabilization and activation of the enzyme carnosinase, which combine with different metal cations to play different biochemical roles (Babizhayev et al., 1994). Previous studies noted that carnosine binds to different metal cations in different ways, but among these, Cu^2+^‐carnosine complex and Zn^2+^‐carnosine complex have been the most well researched (Baran, 2000).

##### The copper‐carnosine complex

Copper is involved in the regulation of inhibitory synaptic transmission, while carnosine binds to copper to reverse these effects (Trombley et al., 1998). The Cu^2+^‐carnosine complex also has similar activity to SOD—preventing the production of superoxide in the brain under oxidative stress, suggested to be an efficient treatment for neurodegenerative diseases (Kohen et al., 1991).

##### The zinc‐carnosine complex

Zinc has similar effects to copper in the CNS, inhibiting N‐methyl‐d‐aspartate receptor and gamma‐aminobutyric acid receptor‐modulated behavior in the brain (Blakemore & Trombley, 2017). The zinc‐chelating action of carnosine may prevent these declines, exerting a neuroprotective role in cognitive‐related diseases (Kohen et al., 1991). A systematic review showed that zinc increases neurotoxicity in AD and vascular dementia. Carnosine reduced endoplasmic reticulum stress through antioxidant and anti‐crosslink activities, as well as through zinc chelation (Kawahara et al., 2018). Meanwhile, the Zn^2+^‐carnosine complex (also called the polaprezinc) has been shown to exert a multiple widespread functions including anti‐ulcer, anti‐*Helicobacter pylori*, healing promotion, anti‐liver fibrosis, and can attenuate gastric mucosa injury, ulcerative colitis, taste disorders, and chronic obstructive pulmonary disease (Li et al., 2021). Additionally, polaprezinc has known anti‐inflammatory and antioxidant activities in chronic inflammatory diseases. Pretreatment of cells with polaprezinc promotes the dissociation of Nrf2 from Keap1 and subsequently activates the Nrf2 signaling pathway, leading to the induction of HO‐1 expression. This then inhibits the activation of the NF‐κB signaling pathway induced by lipopolysaccharide, suppressing the production of pro‐inflammatory mediators (Ooi et al., 2016, 2017).

#### 2.1.4 Anti‐glycation

**FIGURE 3:**
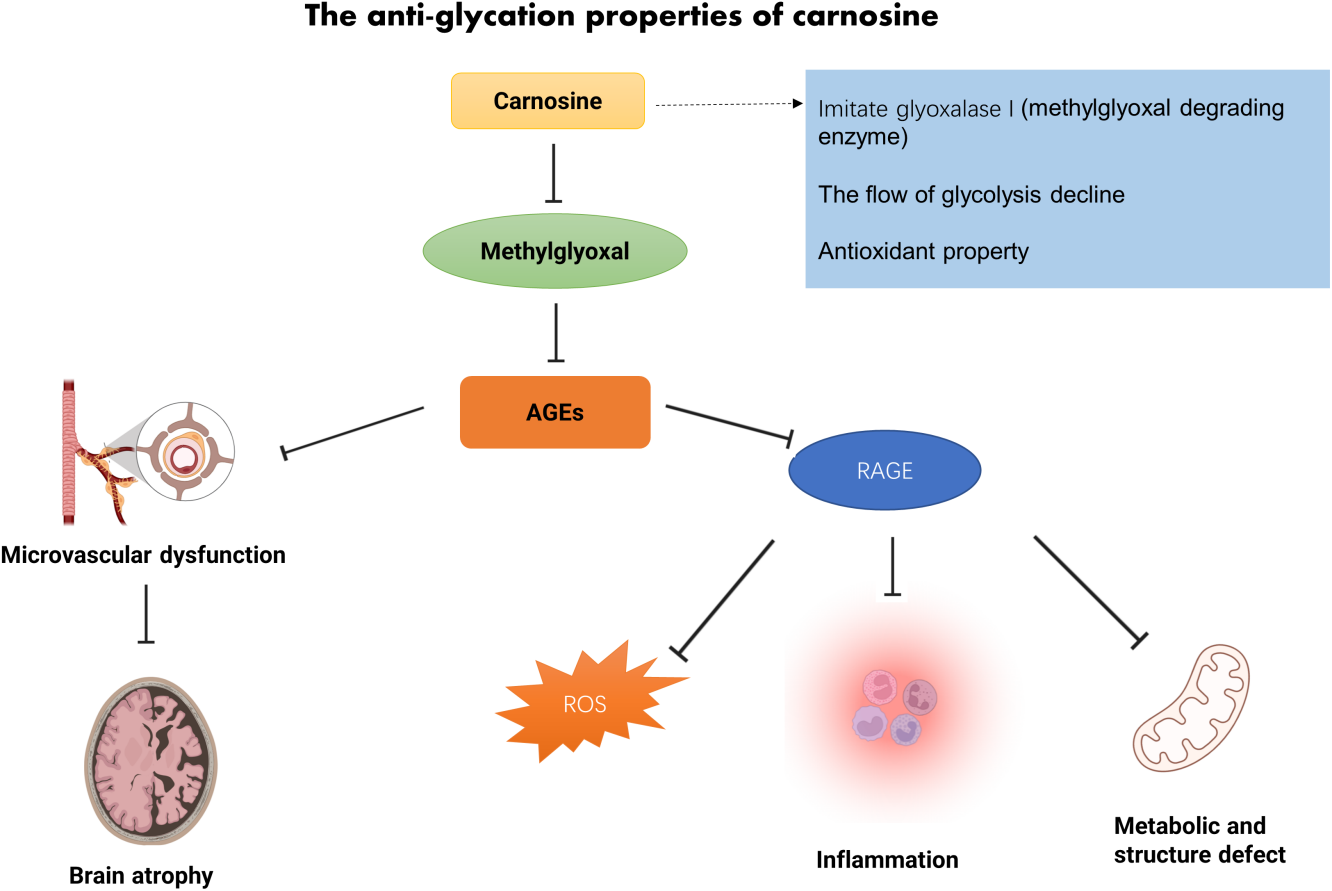
The anti‐glycation of carnosine. AGEs, advanced glycation end products; RAGE, receptor of advanced glycation end product; ROS, generate reactive oxygen species.

Elevated concentrations of acetaldehyde, methylglyoxal, and 3‐deoxyglucose in the plasma are essential factors contributing to AGE accumulation (Almajwal et al., 2020; Nowotny et al., 2015). AGE accumulation alters protein function, resulting in mitochondrial dysregulation, enhancing ROS activation of RAGEs to induce downstream pathogenic cascades, which are positively associated with the development of cancer, neurodegenerative diseases, stroke, and diabetic complications (Monnier et al., 2005; Rabizadeh et al., 2023). Previous studies have shown that carnosine has anti‐glycation properties, suppressing formation of AGEs (Faith Aydin et al., 2017; Pfister et al., 2011). In T2DM patients, carnosine supplementation reduces fasting glucose, serum triglycerides, and AGEs, but with no significant change in soluble RAGE (Houjeghani et al., 2018). In animal models of T2DM, carnosine treatment decreased the level of AGEs in the serum and kidneys, suggesting carnosine may prevent the process of T2DM and diabetic nephropathy (Faith Aydin et al., 2017). However, other research shows that carnosine attenuates retinal vascular damage without changing the production of ROS and AGEs as well as the levels of N(6)‐carboxymethyllysine (a marker of AGEs) and methylglyoxal after oral supplementation with carnosine in rat models of diabetic retinopathy, suggesting other physiological effects beyond its antioxidative roles (Pfister et al., 2011). One possible explanation is that sustained severe hyperglycemia over a period of 3 months may result in a higher oxidative burden, potentially exhausting the protective capacity of carnosine against oxidative stress and hyperglycemia‐induced AGE accumulation (Riedl et al., 2011). In vitro, carnosine also inhibits AGE formation in renal cells and peritoneal mesothelial cells (Alhamdani et al., 2007; Weigand et al., 2018), with multiple mechanisms are thought to underpin this. Carnosine might imitate glyoxalase I activity (methylglyoxal degrading enzyme) to diminish methylglyoxal levels due to its imidazole ring (Battah et al., 2002). Alternatively, during glycolysis, glucose is transformed into methylglyoxal, promotes AGE formation, with carnosine reducing the rate of glycolysis and hence suppressing AGE levels (Nelson & Cox, 2000). Finally, oxidative stress is also an important factor in promoting the production of AGEs, making the antioxidant effect of carnosine a likely inhibitor of AGE formation (Figure 3) (Ghodsi & Kheirouri, 2018).

The antiglycation properties of carnosine may also impart protective benefits in the brain, potentially preventing cognitive decline. In the brain, accumulation of AGEs results in microvascular dysfunction, leading to reduced cerebral blood flow and abnormal atrophy of the cortex (Rodriguez et al., 2009). Additionally, AGEs impair memory function by enhancing ROS production following ligation of RAGEs, reducing neuronal glucose consumption, and neuronal mitochondrial activity in diabetes (Jiang et al., 2022). Conversely, carnosine may alleviate cognitive impairment by reversing the upregulation of RAGEs caused by a high‐fat diet in animal models of AD (Herculano et al., 2013). Thus, carnosine‐mediated inhibition of AGEs and RAGEs is a potential target for alleviating or preventing diabetes‐induced cognitive impairment (Figure 3), though mechanistic studies are required to confirm this.

## 3 THE EFFECT OF CARNOSINE ON COGNITIVE‐RELATED DISEASES

Prior studies have shown that exogenous carnosine can pass the blood–brain barrier and activate glial cells to secrete neurotrophins including brain‐derived neurotrophic factor and nerve growth factor (Jin et al., 2005). In addition, several studies show that carnosine is an effective neuroprotector and improve cognitive function in cerebral damage including neurodegenerative diseases and ischemic injuries in human (Berezhnoy et al., 2019; Hata et al., 2019; Kim et al., 2021).

### 3.1 Effect of carnosine on AD

AD is the most common form of dementia worldwide with at least 50 million people affected globally (Guzman‐Martinez et al., 2021). The disease is pathologically characterized by the deposition of amyloid and phosphorylated tau proteins throughout the brain. It is thought that the pathology starts in the medial temporal cortex and spreads throughout the rest of the brain (de Flores et al., 2022). Carnosine as a supplement or treatment protects against cognitive impairment in dementia and AD. In humans, the level of serum β‐alanine (reflecting intakes of carnosine) is negatively associated with the risks of all‐cause dementia and AD, suggesting that carnosine might prevent the development of dementia (Hata et al., 2019). Additionally, supplementing with anserine/carnosine was found to provide protective effects against cognitive decline in 54 individuals with MCI, particularly in those who are APOE4 positive (a key genetic predictor of dementia) (Masuoka et al., 2019). After 13 weeks of carnosine supplement, auditory long‐term memory, abstract thinking, and constructional praxis are improved in the older adults in comparison to a placebo group (Szczesniak et al., 2014). A similar study shows that carnosine enhances verbal memory and inhibits the decrease of brain blood flow and is correlated with suppression of the inflammatory chemokine CCL24 in older people without dementia (Katakura et al., 2017). In animal studies, treatment with carnosine decreases the AGEs level and oxidative stress in brain of d‐galactose‐induced aging changes in rats (Aydin et al., 2018). Carnosine supplementation increases the steady‐state levels in the brain, improves dendritic spine density and cognitive impairment induced by aging, indicating that supplementation restores the antioxidative activity of endogenous carnosine and reduces neurodegeneration in rats (Banerjee et al., 2021). Carnosine also reduces the intraneuronal accumulation of Aβ and improves mitochondrial dysfunctions in AD mice (Corona et al., 2011). The mitochondrial cascade hypothesis proposes that mitochondrial function influences the expression, processing, and accumulation of Aβ (Swerdlow, 2023). Therefore, carnosine emerges as a promising avenue for addressing AD and associated cognitive impairments, exhibiting potential in both human and animal studies.

### 3.2 Effect of carnosine in cerebral ischemic injury‐induced cognitive impairment

Carnosine has been shown to be an effective neuroprotector in cerebral ischemic injury (Kim et al., 2021; Pekcetin et al., 2009). Tight junction (TJ) protein is a major component of the blood–brain barrier and plays an important role in maintaining its overall integrity. Following administration of carnosine, ischemia‐mediated degradation of TJ protein is hindered, the activity of matrix metalloproteinases are reduced, and infarcted volume and edema are diminished in animal models of ischemic stroke (Kim et al., 2021). Carnosine also plays a protective role in the postischemic period. Treatment with carnosine provides marked protection against neurologic symptoms, and mortality is decreased through attenuation of oxidative stress after the ischemic episode (Dobrota et al., 2005). Pretreatment with carnosine also has a significant protective effect on hypoxia‐ischemia‐induced cognitive deficits, reducing infarct volume, and promoting spatial learning and memory through inhibition of apoptosis and enhanced antioxidation in rats (Zhang et al., 2011). Conversely, other research shows that carnosine does not improve spatial learning, but alleviates oxidative stress and inhibits neuronal apoptosis in ischemic rats (Pekcetin et al., 2009). In vascular dementia caused by chronic hypoperfusion, carnosine attenuates white matter lesions and cognitive deficits through a reduced activation of microglia and astrocytes decreasing reactive ROS and inflammatory mediator production (Ma et al., 2015). Carnosine also exerts a neuroprotective role by modulating histaminergic, improving antioxidation, inflammatory response, and anti‐acetylcholinesterase (AChE) actions in rats with bilateral common carotid artery occlusion‐induced vascular dementia to improve memory (Tiwari et al., 2018). Therefore, carnosine may has a positive impact on the cognitive impairment caused by cerebral ischemic injury, again associated with its antioxidant, anti‐inflammation, and antiapoptotic properties.

## 4 THE EFFECT OF CARNOSINE ON DIABETES

### 4.1 Effect of carnosine on blood glucose

Carnosine supplementation has been shown to reduce the risk of T2DM and to lower blood glucose in patients with T2DM by increasing concentrations of glucagon‐like peptide‐1 and activity of anti‐dipeptidyl peptidase‐4 activity which promote insulin secretion (Vahdatpour et al., 2019). Meanwhile, carnosine supplementation in overweight and obese individuals reduces serum adipokine concentrations involved in glucose metabolism, suggesting potential benefits in preventing T2DM (Baye et al., 2018). Chronic hyperglycemia is the key factor in the development of T2DM, and leads to generation of hydrogen peroxide and ketoaldehydes in the presence of transition metals, accelerating the production of AGEs (Vargas‐Sanchez et al., 2019). AGEs induce cell damage due to increased oxidative stress and activate pro‐inflammatory signaling pathways, such as NF‐κB (Li et al., 2018). Carnosine has been shown to suppress hyperglycemia in patients with diabetes and in animal models of diabetes (Aydin et al., 2017; de Courten et al., 2016; Houjeghani et al., 2018; Matthews et al., 2021; Nagai et al., 2003). For example, in humans, carnosine reduced glucose levels after an oral glucose tolerance test compared to placebo (de Courten et al., 2016), and it improved fasting glucose, HbA1c, and AGEs in T2DM patients (Houjeghani et al., 2018). In mouse models, as in humans, carnosine reduced the accumulation of serum AGEs in high‐fat and low‐dose streptozotocin (STZ)‐induced diabetic rats (Aydin et al., 2017). Additionally, dietary carnosine inhibits the level of blood glucose by the modulation of autonomic nerves in hyperglycemic rats (Nagai et al., 2003). A recent meta‐analysis found that carnosine supplementation decreases fasting glucose and HbA1c in humans and rodents (Matthews et al., 2021). In vitro, carnosine is an effective scavenger of reactive oxygen and nitrogen species in pancreatic β‐cells and promotes insulin secretion and glucose uptake in skeletal muscle cells (Cripps et al., 2017).

### 4.2 Effect of carnosine on insulin

Insulin resistance underpins many metabolic disorders including diabetes, making the improvement of insulin sensitivity a key therapeutic priority (Shazmeen et al., 2021; Yaribeygi et al., 2019). Prior studies have shown that carnosine attenuates blood glucose by increasing C‐peptide and insulin secretion from pancreatic β‐cells (Albrecht et al., 2017). Consistent with these findings, carnosine promotes insulin secretion in β‐cells and primary islets, as well as reversing the damaging suppression of insulin secretion caused by long‐term exposure to high levels of glucose in vitro (Cripps et al., 2017). Carnosine also increases both insulin‐related mRNA and protein levels in pancreatic tissue of T1DM mice, suggesting that carnosine can protect the insulin‐producing β cells (Barca et al., 2018; Vahdatpour et al., 2019).

In addition, insulin plays a critical role in neuronal function through the PI3K/Akt and Ras/mitogen‐activated kinase (MAPK) signaling pathways (Ko et al., 2023; Sedzikowska & Szablewski, 2021). Through the insulin receptor substrate (IRS)/Akt signaling pathways, insulin increases neurite outgrowth, regulates synaptic plasticity (long‐term potentiation and long‐term depression), facilitates dendritic spine formation and promotes development of excitatory synapses, and suppresses neuron apoptosis (Arnold et al., 2018; Kim & Han, 2005; Ozcaliskan Ilkay et al., 2023). The MAPK signaling pathways include extracellular signal‐regulated kinases 1 and 2 (ERK1/2), p38, and JNKs, which are involved in cell growth, survival, and gene expression to promote memory formation (Arnold et al., 2018). Chronic hyperglycemia negatively influences brain function, leading to reduced cognitive function and impaired mood, correlating to insulin resistance in neurons (Maciejczyk et al., 2019). Previous research demonstrates that carnosine reduces the level of Akt in glioblastoma cells (Oppermann et al., 2019), and relieves PMA‐induced Akt phosphorylation in macrophages, providing evidence for a mechanism of activity in the brain (Caruso, Fresta, Fidilio, et al., 2019). In addition, carnosine decreases the extent of nervous system injury by reducing the transformation time profile of ERK1/2 activation, preventing JNK activity, and mediating the MAPK signaling pathway, which effectively improves the survival of neurons (Cheng et al., 2011; Kulebyakin et al., 2012). Taken together, the research suggests that carnosine is involved in mediating PI3K/Akt and MAPK signaling pathways in neuronal cells.

### 4.3 Effect of carnosine in diabetes‐induced cognitive impairment

Recent data shows a close association between T2DM and dementia (Rad et al., 2018; Tumminia et al., 2018). T2DM enhances the risk of AD through acceleration of Aβ accumulation, and reduction in its clearance, due to insulin resistance (Rad et al., 2018). Meanwhile, T2DM and AD share critical characteristics of CNS change, including brain insulin resistance, Aβ accumulation, tau hyperphosphorylation, cerebral microvascular dysfunction, neuroinflammation, and oxidative stress (Huang et al., 2020; Lu et al., 2021; Tumminia et al., 2018). Aβ accumulation and hyperphosphorylated tau protein also imply the accumulation of extracellular neuritic plaques and fibrils and intracellular neurofibrillary tangles which are the main contributors to dementia in AD (de Flores et al., 2022).

Carnosine may play a protective role in the cognitive deficit induced by diabetes. In rat neuronal cultures, carnosine demonstrates neuroprotective effects against Aβ1–42‐induced toxicity (Distefano et al., 2022). This protective mechanism is attributed to the enhancement of insulin‐degrading enzyme activity by carnosine, leading to increased degradation of long substrates such as insulin and Aβ peptides (Distefano et al., 2022). Notably, the insulin‐degrading enzyme involves in reduction of Aβ accumulation in AD and diabetic cognitive impairment (Tian et al., 2023). Consequently, the neuroprotective effects of carnosine may hold therapeutic implications for improving cognitive outcomes in patients with diabetes.

In vivo study, carnosine is reduced in the brain of animals with STZ‐induced diabetes, with an associated decrease in CARNS1 and key transport gene (Slc15a2/Pept2) mRNA as well as an upregulation of intracellular carnosine dipeptidase (Cndp2) (Barca et al., 2018). Interestingly, treatment with exogenous carnosine in diabetic mice partially ameliorates these changes, suggesting that it can partially inhibit the STZ‐induced effects (Barca et al., 2018). Functionally, carnosine treatment attenuates learning and memory dysfunction in T1DM rats, with potential mechanisms including reduction in oxidative stress and neuroinflammation by Nrf2/HO‐1 and NF‐kB signaling pathways, reducing astrogliosis, and AChE activity (Ahshin‐Majd et al., 2016). In a T2DM mice model, carnosine also relieves cognitive impairment and oxidative stress damage by improving the expression of sirtuin 6 and suppressing endoplasmic reticulum stress (Peng et al., 2022). In rat models of T2DM, carnosine may relieve mild cognitive deficits by mediating oxidative stress, regulating Akt/mTOR signaling pathway, and mitigating autophagy in the hippocampus (Ndolo et al., 2023).

## 5 CONCLUSION

Cognitive impairment represents an important cause of a reduction in quality of life and an enhanced economic burden among diabetic patients and their caregivers. The presented human and animal evidence proposed that alternation in neuronal damage resulting in the abnormal insulin signaling pathway, oxidative stress, neuroinflammation, and AGEs accumulation act as leading factors in the development and progression of cognitive impairment induced by T2DM. The mechanisms of neuronal injury converge upon the main four factors, and this appears as a therapeutic target in intervention. Carnosine is a substance naturally produced by the body and is detected in the muscle and brain of humans. Also, the content of carnosine could be exogenously supplied from natural diets such as beef and fish with nontoxic and no side effects (Aliani et al., 2013). While consuming enough beef and fish to achieve the desired carnosine intake might not be feasible, making carnosine supplementation is a more viable option. Additionally, carnosine is produced naturally in the body, carnosinase activity has been shown to increase with age, leading to a reduced concentration of available carnosine within the CNS (Bellia et al., 2009). This means that carnosine has potential as a promising oral formulation of multi‐protective therapy for the prevention or treatment of diabetes and neurodegenerative diseases. Specifically, through its antioxidant, anti‐inflammatory, and anti‐glycation properties, carnosine alleviated cognitive dysfunction, mediated insulin resistance, delayed oxidative damage, downregulated inflammatory cytokines, and inhibited the formation of AGEs. Meanwhile, the neuroprotective role of carnosine has been demonstrated in T1DM‐induced cognitive impairment by regulating oxidative stress and neuroinflammation (Ahshin‐Majd et al., 2016). The accumulative evidence supports the multiple roles of carnosine as the efficient protective agent for delaying the onset and progression of T2DM and treating T2DM‐induced complications. Therefore, we suggest that carnosine may affect cognitive function positively in T2DM when it is prescribed as a dietary supplement.

## AUTHOR CONTRIBUTIONS

**Qian Wang:** Visualization (lead); writing – original draft (lead); writing – review and editing (equal). **Nicholas Tripodi:** Writing – review and editing (equal). **Zachary Valiukas:** Writing – review and editing (equal). **Simon M. Bell:** Writing – review and editing (equal). **Arshad Majid:** Writing – review and editing (equal). **Barbora de Courten:** Writing – review and editing (equal). **Vasso Apostolopoulos:** Supervision (equal); writing – review and editing (equal). **Jack Feehan:** Conceptualization (lead); supervision (equal); writing – review and editing (equal).

## FUNDING INFORMATION

The authors declare not receiving external funds for the research and/or publication of this article.

## CONFLICT OF INTEREST STATEMENT

The authors declare no conflicts of interest.

## ETHICS STATEMENT

This study is a review article and did not involve direct experimentation on any animals and humans and no ethical approval was required.

## Data Availability

All data that support the findings of this study are included in this review article.
